# Validation of Reference Genes for Quantitative Real-Time PCR Normalization in *Ananas comosus* var. *bracteatus* During Chimeric Leaf Development and Response to Hormone Stimuli

**DOI:** 10.3389/fgene.2021.716137

**Published:** 2021-10-21

**Authors:** Meiqin Mao, Yanbin Xue, Yehua He, Xuzixing Zhou, Hao Hu, Jiawen Liu, Lijun Feng, Wei Yang, Jiaheng Luo, Huiling Zhang, Xi Li, Jun Ma

**Affiliations:** ^1^ College of Landscape Architecture, Sichuan Agricultural University, Chengdu, China; ^2^ College of Horticultural Biotechnology, South China Agricultural University, Guangzhou, China

**Keywords:** Ananas comosus var bracteatus, RT-qPCR, reference genes, tissue, chimeric leaf development, hormone stimuli, gene expression

## Abstract

Reverse transcription quantitative real-time PCR (RT-qPCR) is a common way to study gene regulation at the transcriptional level due to its sensibility and specificity, but it needs appropriate reference genes to normalize data*. Ananas comosus var. bracteatus*, with white-green chimeric leaves, is an important pantropical ornamental plant. Up to date, no reference genes have been evaluated in *Ananas comosus* var. *bracteatus*. In this work, we used five common statistics tools (geNorm, NormFinder, BestKeeper, ΔCt method, RefFinder) to evaluate 10 candidate reference genes. The results showed that *Unigene.16454* and *Unigene.16459* were the optimal reference genes for different tissues, *Unigene.16454* and zinc finger ran-binding domain-containing protein 2 (*ZRANB2*) for chimeric leaf at different developmental stages, isocitrate dehydrogenase [NADP] (*IDH*) and triacylglycerol lipase SDP1-like (*SDP*) for seedlings under different hormone treatments. The comprehensive results showed *IDH*, pentatricopeptide repeat-containing protein (*PPRC*), *Unigene.16454,* and caffeoyl-CoA O methyltransferase 5-like (*CCOAOMT*) are the top-ranked stable genes across all the samples. The stability of glyceraldehyde-3-phosphate dehydrogenase (*GAPDH*) was the least during all experiments. Furthermore, the reliability of recommended reference gene was validated by the detection of porphobilinogen deaminase (*HEMC*) expression levels in chimeric leaves. Overall, this study provides appropriate reference genes under three specific experimental conditions and will be useful for future research on spatial and temporal regulation of gene expression and multiple hormone regulation pathways in *Ananas comosus* var. *bracteatus*.

## Introduction

*Ananas comosus* var. *bracteatus* is a monocotyledon from the Bromeliaceae family that originates from tropical and subtropical areas of South America (Baker and Collins, 1939; Duval et al., 1997). Historically, it has been cultivated for fiber and fruit juice and is now a popular pantropical ornamental plant (Baker and Collins, 1939; Duval et al., 1997; Ma et al., 2015). In addition, *Ananas comosus* var. *bracteatus* is an excellent material for studying leaf albino mechanisms due to its white-green chimeric leaf (Mao et al., 2020). The formation of leaf albino mutants involves multiple biological processes, including photosynthesis, chlorophyll biosynthesis, chloroplast development, and hormone signaling pathways (Córdoba et al., 2016; Gao et al., 2016; Zhang et al., 2018; Zuo et al., 2019; Liu et al., 2020). Thus, complicated molecular regulatory networks of genetics, signaling, and metabolic mechanisms may exist in *Ananas comosus* var. *bracteatus*. Gene expression analysis is a popular and powerful tool to elucidate these complicated molecular regulatory networks (Bustin et al., 2005).

Reverse transcription-quantitative real-time PCR (RT-qPCR) is the most common method for analyzing gene expression level and validating the results obtained from other methods such as RNA-Seq (Bustin, 2002; Gachon et al., 2004; Bustin et al., 2005; Erickson et al., 2009; Kozera and Rapacz, 2013; Takamori et al., 2017). However, the prerequisite for avoiding inaccurate results by RT-qPCR is to select reliable reference genes for standard correction (Derveaux et al., 2010). Generally speaking, an ideal reference gene should be stably expressed independently of any experimental conditions (Kozera and Rapacz, 2013). Housekeeping genes, such as glyceraldehyde-3-phosphate dehydrogenase (*GAPDH*), actin (*ACT*), elongation factor-1a (*EF1α*), and 18S ribosomal RNA (*18S*), play essential roles in basic cellular progress, suggesting that they are stably expressed (Nicot et al., 2005; Garg et al., 2010; Maroufi et al., 2010). Based on the assumption that housekeeping genes are expressed at constant levels under all conditions, they are usually used as reference genes without verifying their stability. However, several studies indicated that the transcription stability of housekeeping genes is affected by internal and external conditions (Czechowski et al., 2005; Gutierrez et al., 2008; Sgamma et al., 2016; Wan et al., 2017). Therefore, no single reference gene, including housekeeping genes, is appropriate in all experimental conditions. As a result, reference genes must be systematically screened prior to specific experimental conditions (Cheng et al., 2017).

Previous studies on *Ananas comosus* var. *comosus* recommended several reference genes for gene expression analysis in pineapple plants, and mainly assayed reproductive organ development and abiotic/biotic stress conditions (Ma et al., 2012; Chen et al., 2019; Jin et al., 2020). For instance, housekeeping genes *GAPDH* and *18S* are considered reference genes during pineapple organ development. In addition, several novel reference genes have been found based on pineapple genome and transcriptome databases. This method has been successfully applied to several plants, such as *Arabidopsis thaliana*, *Oryza sativa*, and *Solanum lycopersicum* (Czechowski et al., 2005; Narsai et al., 2010; Müller et al., 2015). Given this genetic background and biological process, the reference genes in *Ananas comosus* var. *comosus* may not be directly applied in *Ananas comosus* var. *bracteatus* (Galli et al., 2015; Jian et al., 2016; Wang and Lu, 2016). Therefore, systematic selection of reliable reference genes should be performed in *Ananas comosus* var. *bracteatus*.

In this study, we evaluated the expression stability of 10 candidate reference genes in different tissues (root, leaf, stem, and flesh), chimeric leaf at different developmental stages, and seedlings under different hormone treatments [Indole-3-acetic Acid (IAA), Trans-zeatin (T-z), Gibberellin A3 (GA3), Abscisic Acid (ABA), Brassinolide (BR)]. Gene expression stability was assayed by four common statistics tools including geNorm, NormFinder, BestKeeper, and ΔCt method (Vandesompele et al., 2002; Andersen et al., 2004; Pfaffl et al., 2004; Silver et al., 2006), then the comprehensive rank was obtained by RefFinder (Xie et al., 2012). Finally, porphobilinogen deaminase (*HEMC*) was analyzed to compare stable and unstable reference genes. Our results provide appropriate reference genes under three specific experimental conditions and will be useful for future research on the spatial and temporal regulation of gene expression and multiple hormone regulation pathways in *Ananas comosus* var. *bracteatus*.

## Materials and Methods

### Plant Materials

*Ananas comosus* var. *bracteatus* was obtained from a garden in Zhanjiang (Guangdong, China) and cultivated in a greenhouse at Sichuan Agricultural University (Sichuan, China). Green tissue culture plantlets were derived from stem explants of *Ananas comosus* var. *bracteatus.* The stem explants were sterilized and then transplanted on MS medium containing 6-BA (3 mg/L) and NAA (2 mg/L). After 1 month, callus was transplanted on MS medium containing 6-BA (0.5 mg/L) and NAA (3 mg/L). Then, green tissue culture plantlets were planted in plastic pots.

There are three experimental sets in this study (Table 1). In experimental set 1, chimeric leaves at different developmental stages were collected. Chimeric leaf development stages (S) were as follows: stage 1 (S1, no chlorophyll is visible on the leaf); stage 2 (S2, chlorophyll becomes visible on leaf center); stage 3 (S3, two thirds of the leaf are fully pigmented by chlorophyll) (Additional File 1: Supplementary Figure S1). In experimental set 2, green tissue culture plantlets with 10–12 expanded leaves were treated with different hormones. Then, leaves were collected after 4 and 24 h hormone treatments, respectively. In experimental set 3, roots, stem, flesh, white tissues, and green tissues were collected separately. All samples were stored at −80°C until RNA extraction.

**TABLE 1:** Samples used in this study.

Experimental set	Sample name	Sample description
Set 1	S1W	White parts of chimeric leaves at stage 1
—	S1G	Green parts of chimeric leaves at stage 1
—	S2W	White parts of chimeric leaves at stage 2
—	S2G	Green parts of chimeric leaves at stage 2
—	S3W	White parts of chimeric leaves at stage 3
—	S3G	Green parts of chimeric leaves at stage 3
Set 2	CK-4	Tissue culture plantlets treated with water on 4th h
—	CK-24	Tissue culture plantlets treated with water on 24th h
—	IAA-3	Tissue culture plantlets treated with 10 μM IAA on 4th h
—	IAA-24	Tissue culture plantlets treated with 10 μM IAA on 24th h
—	ABA-4	Tissue culture plantlets treated with 100 μM ABA on 4th h
—	ABA-24	Tissue culture plantlets treated with 100 μM ABA on 24th h
—	GA3-4	Tissue culture plantlets treated with 100 μM GA3 on 4th h
—	GA3-24	Tissue culture plantlets treated with 100 μM GA3 on 24th h
—	BR-4	Tissue culture plantlets treated with1 μM BR on 4th h
—	BR-24	Tissue culture plantlets treated with 1 μM BR on 24th h
—	T-z-4	Tissue culture plantlets treated with 20 μM T-z on 4th h
—	T-z-24	Tissue culture plantlets treated with 20 μM T -z on 24th h
Set 3	Root	—
—	Stem	—
—	Fresh	—
—	White tissues	White parts of chimeric leaves at stage 3
—	Green tissues	Green parts of chimeric leaves at stage 3

### RNA Extraction and cDNA Synthesis

Total RNA was extracted using LABGENE^TM^ Plant RNA Isolation Kit (Cat. No. LB1111, LABGENE Biotechnology Co., Ltd., Chengdu, China). The concentration and purity of extracted RNA were measured using NanoDrop 2000 spectrophotometer (Thermo Scientific, Waltham, MA, United States), and 1.2% (w/v) agarose gel electrophoresis was performed to assess the RNA integrity. When A260/A280 ratio was between 1.8 and 2.2, and A260/A230 ratio was higher than 1.8, RNA can be used for further analysis. cDNA was synthesized from 1.0 μg RNA using *Evo M-MLV* RT Kit whit gDNA Clean for qPCR II (Code No. AG11711, Accurate Biotechnology Co., Ltd., Wuhan, China), then stored at −20°C until use.

### Candidate Gene Selection and Primer Design

The 10 candidate reference genes included two previously assessed pineapple reference genes, *GAPDH*, *18S*, as well as 8 novel candidate reference genes chosen from the transcriptome of *Ananas comosus* var. *bracteatus* (NCBI accessions: PRJNA564223), *Unigene.16454*, caffeoyl-CoA O methyltransferase 5-like (*CCOAOMT*), triacylglycerol lipase SDP1-like (*SDP*), *Unigene.16459*, zinc finger ran-binding domain-containing protein 2 (*ZRANB2*), pentatricopeptide repeat-containing protein (*PPRC*), and isocitrate dehydrogenase (NADP) (*IDH*), *Unigene.26260* (Table 2, Additional file 2: Supplementary Table S1). Primer specificity was judged by gel electrophoresis and melting-curve analyses. In addition, the amplification efficiency (E) of the primer was calculated by standard curve.

**TABLE 2:** Description of candidate reference genes.

Gene name	Gene symbol	Accession number	Functions
*Unigene.16454*	*Unigene.16454*	LR828290.1	Unknown function
*Caffeoyl-CoA O methyltransferase 5-like*	*CCOAOMT*	XM_020225704.1	Feruloylated polysaccharides synthesis
*Triacylglycerol lipase SDP1-like*	*SDP*	XM_020244972.1	Hydrolyze triacylglycerols
*Unigene.16459*	*Unigene.16459*	LR828290.1	Unknown function
*Zinc finger Ran-binding domain-containing protein 2*	*ZRANB2*	LR828285.1	Cooperates gene transcription
*Pentatricopeptide repeat-containing protein*	*PPRC*	LR862139.1	RNA-binding protein that functions in both mitochondrion and nucleus
*Isocitrate dehydrogenase (NADP)*	*IDH*	XM_020224677.1	Response to oxidative stresses
*Unigene.26260*	*Unigene.26260*	LR828298.1	Unknown function
*18S ribosomal RNA*	*18S*	—	Cytosolic small ribosomal subunit, translation
*glyceraldehyde 3-phosphate Dehydrogenase*	*GAPDH*	—	Oxidoreductase in glycolysis and gluconeogenesis

### RT-qPCR Analysis

The RT-qPCR was performed with CFX96 Real-time PCR Detection System (Bio-Rad, US) using the SYBR^®^ Green Premix *Pro Taq* HS qPCR Kit (Cat No. AG11701, Accurate Biotechnology Co., Ltd., Wuhan, China). 25 μL reaction consisted 12.5 μL 2X SYBR^®^ Green *Pro Taq* HS Premix, 0.5 μL each primer (10 μM), 1 μL of cDNA, and 10.5 μL dH_2_O. The following RT-qPCR amplification program was 95°C for 30 s, and 40 cycles of 95°C for 5 s, the optimal temperature for each primer for 15 and 15 s for 72°C. The dissociation curve was then generated by melting the amplicon from 60 to 95°C. All RT-qPCR experiments were performed in three biological replicates and three technical replicates. A no-template control and a reverse transcription negative control were also performed.

### Gene Expression Stability Analysis

First of all, the mean Cq value was calculated by three biological replicates to analyze expression levels. Secondly, geNorm, NormFinder, BestKeeper, and ΔCt method were applied to evaluate expression stability (Vandesompele et al., 2002; Andersen et al., 2004; Pfaffl et al., 2004; Silver et al., 2006). Finally, RefFinder online (http://blooge.cn/RefFinder/) was used to obtain comprehensive ranks (Xie et al., 2012).

### Validation of Identified Reference Genes

*HEMC* is a key gene involved in chlorophyll biosynthesis and was used as a target gene to verify the results of our experiment. The most and least stable reference gene, as well as the combination of the two best reference genes, were used respectively to normalize the expression profile of *HEMC* in the green and white parts of the chimeric leaf at different developmental stages. The relative expression was conducted with the comparative 2^−ΔΔCT^ method (Livak and Schmittgen, 2001).

### Gene Expression Analysis of *AbGLK1*

*Golden 2-like*, or *GLK*, transcription factors are known to be involved in chloroplast development and have also been implicated in various hormone signaling pathways. (Fitter et al., 2002; Savitch et al., 2007; Gutiérrez et al., 2008; Waters et al., 2009). The expression profiles of *AbGLK1* during chimeric leaf development and response to hormone stimuli were obtained according to the comparative 2^−ΔΔCT^ method (Livak and Schmittgen, 2001). Sample collections and experiments were performed as described above.

## Results

### Primer Specificity and Amplification Efficiency of the Candidate Reference Genes

**FIGURE 1:**
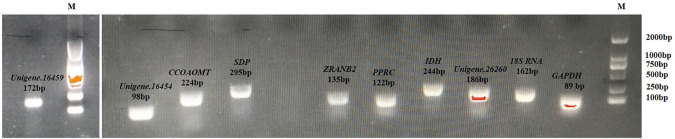
Amplified fragment of 10 candidate genes on 1.2% agarose gel. M represents the DL2000 DNA marker.

**FIGURE 2:**
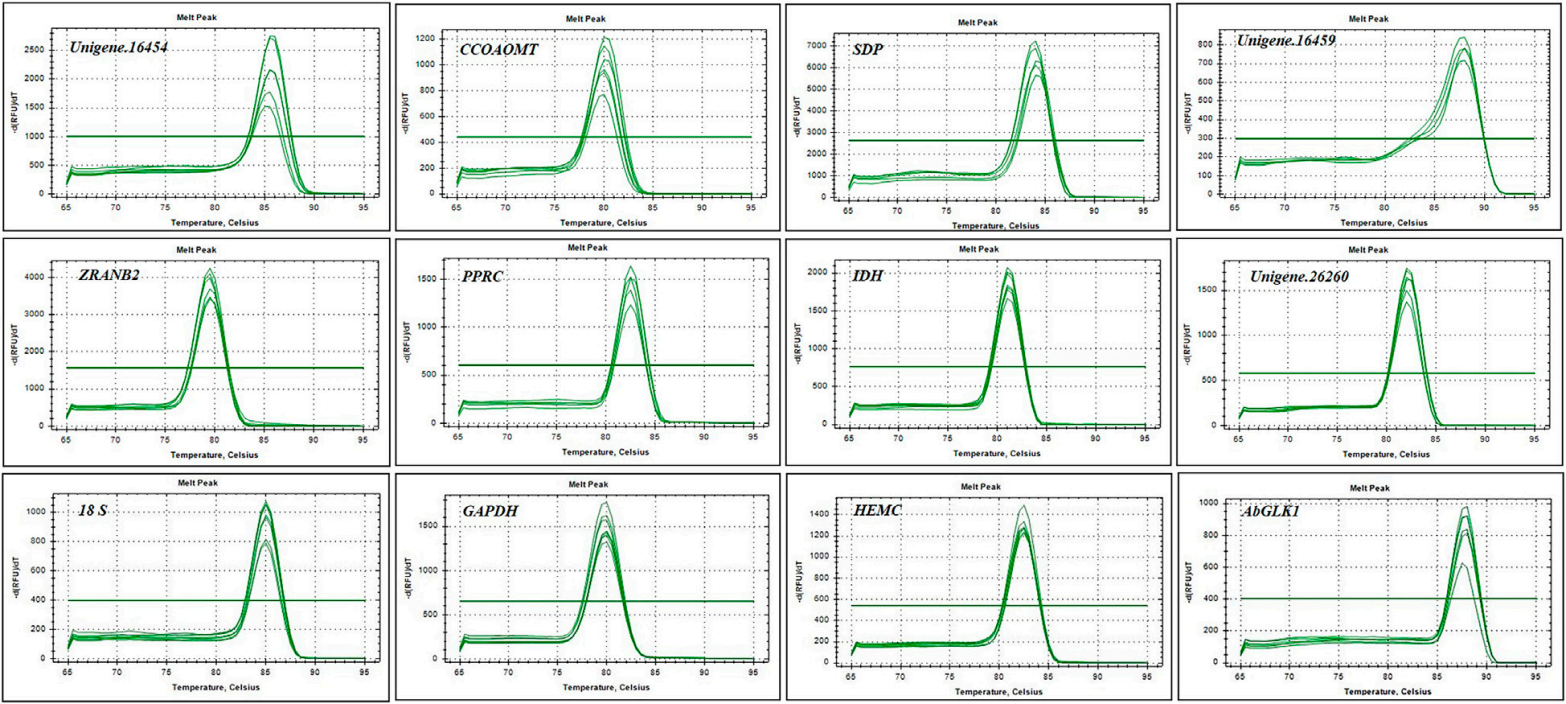
Melting curves of 10 candidate reference genes and two target gene.

Gel electrophoresis and melting curve analysis showed that all primer pairs present a single band and a single peak (Figures 1,2), which validates the specificity of primers. No amplification appeared in the negative control. In addition, the amplification efficiency for each primer pair varied from 90.2% for *SDP* to 108.3% for *CCOAOMT*, and the correlation coefficients (R^2^) were all higher than 0.98 (Table 3). Therefore, these primers were used in further RT-qPCR analysis.

**TABLE 3:** Primer used for quantitative real-time PCR analyses.

Gene symbol	Primer sequence (5'-3')	Length (bp)	Efficiency (%)	R^2^
*Unigene.16454*	TCT​CAC​GCC​CTC​TTT​CTT​CCA	98	107.8	0.993
	GCT​CTA​ACT​CGC​CAC​GCC​TTT			
*CCOAOMT*	AAG​CTG​GGA​TAG​TTC​AAT​GCG	224	108.3	0.990
	TTG​TGG​ACG​CCG​ATA​AAG​AAA			
*SDP*	TGG​GCG​GCG​TAT​TTA​CTG​TGG	295	90.2	0.995
	CCA​AAC​CTA​TCC​TTC​GCC​ATC			
*Unigene.16459*	GGA​TTT​GAA​GCA​TCA​TGG​CAC​A	172	98.5	0.981
	AGC​ACA​ACC​CAC​CTC​ATT​TCG			
*ZRANB2*	AGA​GTG​GCT​GGA​TTG​GTG​CTA	135	92.5	0.990
	CTT​ATT​CAT​ACC​GTC​TTG​CTT​T			
*PPRC*	GAG​TCA​GAC​GAT​CCC​TCT​ACC​A	122	103.3	0.981
	TTG​CGT​TGA​GTT​TCA​CCT​TCC			
*IDH*	GAG​TCT​ATT​CGG​GCC​TTT​GCT	244	92.5	0.993
	CCC​AGA​CAT​ACC​CTC​CCT​CAC			
*Unigene.26260*	GGA​GCT​GCA​TTA​GTA​GGT​GGT	187	92.5	0.997
	AGG​AAG​CGT​GTA​GTG​GTT​TGG			
*18S*	ATGGTGGTGACGGGTGAC	162	104.0	0.994
—	CAG​ACA​CTA​AAG​CGC​CCG​GTA	—	—	—
*GAPDH*	ATA​ACA​GGT​CCA​GCA​TCT​T	89	105.0	0.997
	CCACTCGTTGTCATACCA			
*HEMC*	TCG​CTG​GCT​ATG​CTT​GTC​GTG	138	101.4	0.988
—	CTT​TGC​CCA​TTG​CGA​CCA​TAA	—	—	—
*AbGLK1*	ATT​CGT​TCA​AGC​GGT​GGA​GCA	189	108.5	0.996
—	TAC​ATT​TGC​CGC​CTT​TGG​GTC	—	—	—

### Expression Levels of Candidate Reference Genes

**FIGURE 3:**
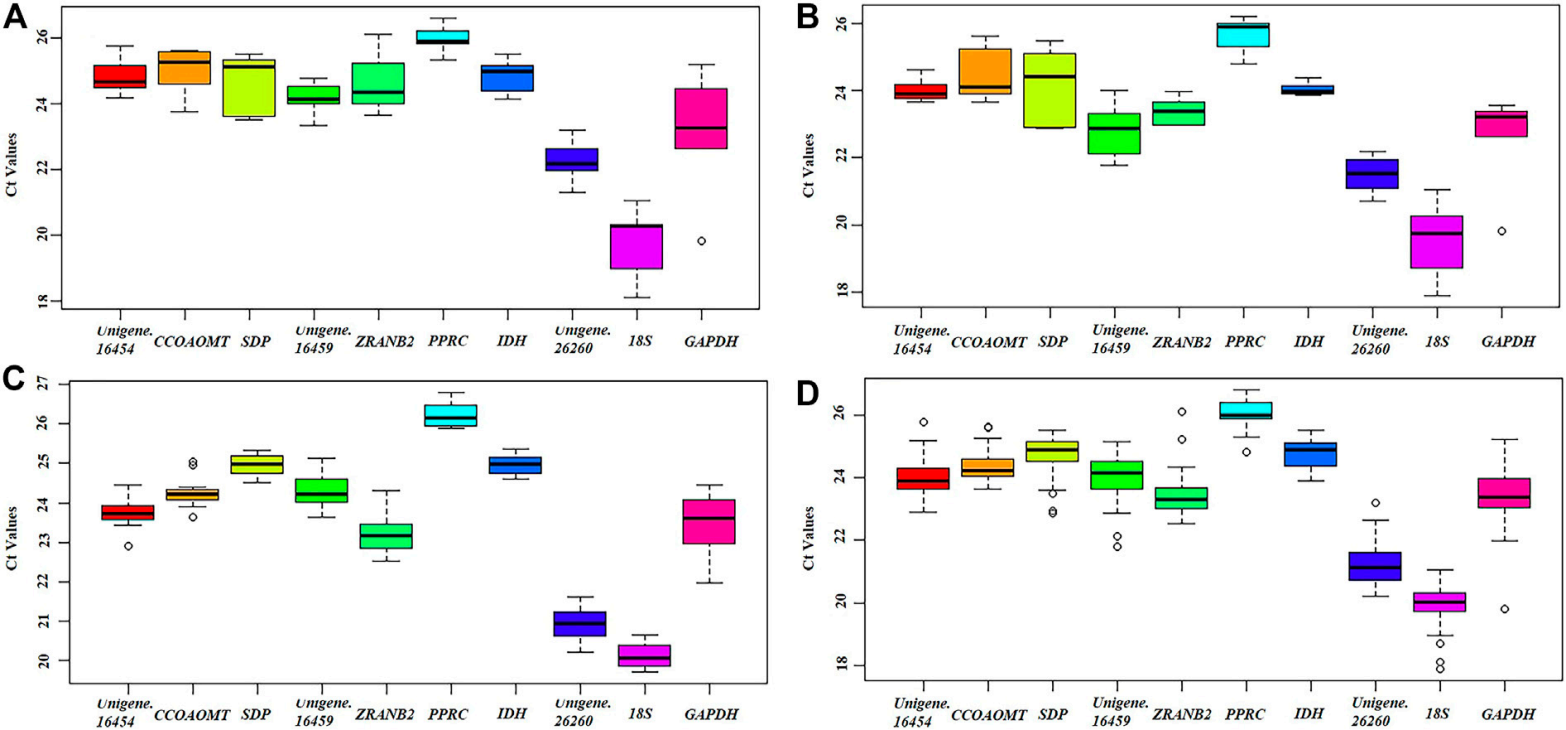
Distribution of Cq values of candidate reference genes in all samples. **(A)** Tissue, **(B)** Developmental stage, **(C)** Hormone treatment, **(D)** Global analysis. Boxplots show the 25th and 75th percentiles, mean, and outliers.

Raw quantification cycle (Cq) values can directly reflect gene expression level, and therefore provide basic data support to evaluate candidate reference genes in tested samples (Yan et al., 2011; Chen et al., 2015). In this study, three specific experimental sets were analyzed individually (Table 1). The raw Cq values of the 10 genes are shown in Additional file 3: Supplementary Table S2 and were plotted directly by Boxplot (Figure 3). In all experimental conditions, *18S* and *PPRC* genes showed the highest and lowest expression levels, respectively. In detail, the Cq means values ranged from 19.74 (*18S*) to 25.96 (*PPRC*) in different tissues, 19.57 (*18S*) to 25.68 (*PPRC*) in chimeric leaf at different developmental stages, 19.83 (*18S*) to 26.03 (*PPRC*) in seedlings under different hormone treatments, and 20.14 (*18S*) to 26.23 (*PPRC*) in all samples. There was no gene to express stably in all experimental conditions, so it was necessary to rank the expression stabilities by using statistical algorithms.

### Expression Stability Analysis by geNorm

GeNorm calculates the average pairwise expression ratio to evaluate expression stability, and genes with lower stability measure (M) values were more stable (Vandesompele et al., 2002). For tissue samples, *Unigene.16454* and *Unigene.16459* (0.236) had the lowest M values. In addition, *Unigene.16454* and *ZRANB2* (0.241) were the most stably expressed genes in the chimeric leaf during different developmental stages. Under hormone treatments, the two most stably expressed genes were *Unigene.16454* and *Unigene.16459* (0.219). Concerning all samples tested, *PPRC* and *IDH* (0.421) were the best reference genes. Notably, *GAPDH* was considered the least stable gene in all experimental conditions (Table 4).

**TABLE 4:** Stability of reference gene expression in each subset.

Experimental conditions	Reference gene	geNorm		Normfinder		BestKeeper		Delta ct		RefFinder	
		S	R	S	R	S	R	S	R	S	R
Tissue	*Unigene.16454*	0.236	1	0.118	1	2.46	4	0.85	1	1.41	1
—	*CCOAOMT*	0.859	7	1.181	8	3.14	6	1.41	8	7.44	8
—	*SDP*	0.737	6	1.020	7	4.29	8	1.29	7	7.24	7
—	*Unigene.16459*	0.236	1	0.118	2	2.05	2	0.88	2	1.68	2
—	*ZRANB2*	0.412	3	0.632	6	4.09	7	1.05	6	5.63	6
—	*PPRC*	0.578	5	0.546	5	1.70	1	1.01	5	3.50	4
—	*IDH*	0.322	2	0.367	4	2.30	3	0.94	3	3.22	3
—	*Unigene.26260*	0.482	4	0.360	3	2.91	5	0.96	4	4.16	5
—	*18S*	0.991	8	1.374	9	5.86	9	1.59	9	9.00	9
—	*GAPDH*	1.202	9	1.959	10	7.88	10	2.05	10	10.00	10
Stage	*Unigene.16454*	0.246	1	0.123	1	1.38	2	0.64	2	1.41	1
—	*CCOAOMT*	0.435	5	0.447	6	3.37	7	0.74	6	6.24	6
—	*SDP*	0.510	6	0.739	7	4.48	8	0.89	7	7.45	7
—	*Unigene.16459*	0.397	4	0.329	5	3.14	6	0.68	4	4.95	5
—	*ZRANB2*	0.246	1	0.123	2	1.90	3	0.63	1	1.57	2
—	*PPRC*	0.621	8	0.741	8	2.09	4	0.96	9	7.54	8
—	*IDH*	0.291	2	0.323	4	0.74	1	0.72	5	2.78	3
—	*Unigene.26260*	0.322	3	0.154	3	2.41	5	0.64	3	3.46	4
—	*18S*	0.553	7	0.771	9	5.27	10	0.92	8	8.24	9
—	*GAPDH*	0.867	9	1.802	10	5.11	9	1.85	10	10.00	10
Hormone	*Unigene.16454*	0.219	1	0.309	6	1.56	5	0.47	4	3.46	3
—	*CCOAOMT*	0.379	5	0.295	4	1.34	3	0.47	5	4.36	6
—	*SDP*	0.283	2	0.228	2	1.11	2	0.44	2	2.21	2
—	*Unigene.16459*	0.219	1	0.304	5	1.68	7	0.47	6	3.94	5
—	*ZRANB2*	0.449	8	0.552	9	1.89	9	0.48	9	9.00	9
—	*PPRC*	0.362	4	0.281	3	1.40	4	0.63	3	3.87	4
—	*IDH*	0.340	3	0.130	1	0.99	1	0.47	1	1.41	1
—	*Unigene.26260*	0.417	7	0.352	8	1.85	8	0.52	8	7.74	8
—	*18S*	0.400	6	0.336	7	1.59	6	0.51	7	6.09	7
—	*GAPDH*	0.523	9	0.767	10	3.22	10	0.82	10	10.00	10
Total	*Unigene.16454*	0.671	3	0.456	2	2.38	4	0.80	2	2.83	3
—	*CCOAOMT*	0.613	2	0.595	5	2.33	3	0.88	4	3.66	4
—	*SDP*	0.794	7	0.653	7	2.99	5	0.91	7	7.24	7
—	*Unigene.16459*	0.769	6	0.591	4	3.38	8	0.88	5	5.96	6
—	*ZRANB2*	0.728	5	0.745	8	3.25	7	0.96	8	7.44	8
—	*PPRC*	0.427	1	0.523	3	1.69	1	0.84	3	1.73	2
—	*IDH*	0.427	1	0.296	1	2.08	2	0.74	1	1.19	1
—	*Unigene.26260*	0.705	4	0.620	6	3.12	6	0.89	6	5.48	5
—	*18S*	0.829	8	0.789	9	3.56	9	1.00	9	8.13	9
—	*GAPDH*	0.918	9	1.129	10	3.89	10	1.27	10	10.00	10

Tissue represents samples from different tissues. Stage represents samples from chimeric leaves at different developmental stages. Hormone represents samples from tissue culture plantlet under different hormone treatments. Total represents samples from the above three experimental conditions. S and R represent the stability values and rank of each method, respectively.

**FIGURE 4:**
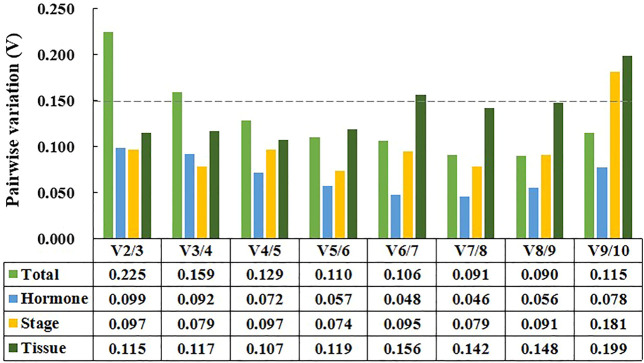
Pairwise variation (Vn/Vn+1) calculated by for geNorm. Tissue represents samples from different tissues. Stage represents samples from chimeric leaves at different developmental stages. Hormone represents samples from tissue culture plantlet under different hormone treatments. Total represents samples from the above three experimental conditions.

In addition, GeNorm can determine the optimal number of reference genes by calculating pairwise variation value (V_n_/V_n +1_, V-value) (Vandesompele et al., 2002). The number of reference genes increased to n+1 when the V-value was higher than 0.15 (Vandesompele et al., 2002). As shown in Figure 4, two reference genes were sufficient for data normalization in tissue samples, developmental stage samples, and hormone treatment samples. For all samples tested, however, the V-value of V2/V3 and V3/V4 were both above 0.15, and thus four reference genes should be selected to normalize gene expression results.

### Expression Stability Analysis by NormFinder

NormFinder has a similar mathematical model to GeNorm, and genes with higher stability values (SV) were considered as less stable (Pfaffl et al., 2004). The most stably expressed genes were *Unigene.16459* (SV = 0.118) and *Unigene.16459* (SV = 0.118) for tissue samples, *Unigene.16454* (SV = 0.123) and *ZRANB2* (SV = 0.123) for developmental stage samples, *IDH* (SV = 0.130) for hormone treatment samples, *IDH* (SV = 0.296) for all samples (Table 4). Like geNorm, *GAPDH* was ranked as the worst reference gene in all experimental conditions (Table 4).

### Expression Stability Analysis by BestKeeper

BestKeeper program evaluates the expression stabilities by the standard deviation (SD) and coefficient variance (CV) (Andersen et al., 2004). The lower CV and SD (CV ± SD) value, the higher stability. As shown in Table 4, *PPRC* (1.35 ± 0.5) and *IDH* (0.60 ± 0.14) were the most stably expressed genes for tissue samples and developmental stage samples, respectively. *IDH* (0.79 ± 0.20) and *SDP* (0.89 ± 0.22) were the two most reliable genes for hormone treatment samples. In addition, *PPRC* (1.34 ± 0.35) and *IDH* (1.67 ± 0.41) were the two most stable reference genes for all tested samples. The SD value of stable expression genes should be lower than the threshold of 1 (Andersen et al., 2004). As shown in Table 4, the SD value of *GAPDH* was higher than 1 in all experimental conditions, suggesting it was an unstable gene.

### Expression Stability Analysis by ΔCt Method

ΔCt method ranks the candidate reference genes based on average standard deviation (SD), which compared the relative expression of “pairs of genes” within each sample (Silver et al., 2006). *Unigene.16454* (0.85) was the most stable reference gene for tissue samples, *ZRANB2* (0.63) for developmental stage samples, and *IDH* (0.47) for hormones treatments samples (Table 4). Concerning all samples tested, *IDH* (0.74) was the best reference gene (Table 4).

### Comprehensive Stability Analysis of Candidate Reference Genes

The four statistical algorithms created different ranking patterns. To obtain a consensus result, a comprehensive analysis based on RefFinder was thus carried out (Xie et al., 2012). Given the optimal number of reference genes, reference genes in three specific experimental conditions were determined in this study. We recommend *Unigene.16454* and *Unigene.16459* as reference genes for tissue samples, *Unigenes.16454* and *ZRANB2* for developmental stage samples, *IDH* and *SDP* for hormone treatment samples, *IDH*, *PPRC*, *Unigene.16454,* and *CCOAOMT* for all samples tested (Table 5).

**TABLE 5:** Best combination of reference genes based on the geNorm and RefFinder.

Tissue		Stage		Hormone		Total	
Most	Least	Most	Least	Most	Least	Most	Least
*Unigene16454*	*GAPDH*	*Unigene16454*	*GAPDH*	*IDH*	*GAPDH*	*IDH*	*GAPDH*
*Unigene16459*	—	*ZRANB2*	—	*SDP*	—	*PPRC*	—
—	—	—	—	—	—	*Unigene16454*	—
—	—	—	—	—	—	*CCOAOMT*	—

Tissue represents samples from different tissues. Stage represents samples from chimeric leaves at different developmental stages. Hormone represents samples from tissue culture plantlet under different hormone treatments. Total represents samples from the above three experimental conditions. Most and least represent the most stable reference gene and the least stable reference gene, respectively.

### Validation of the Recommended Reference Genes

**FIGURE 5:**
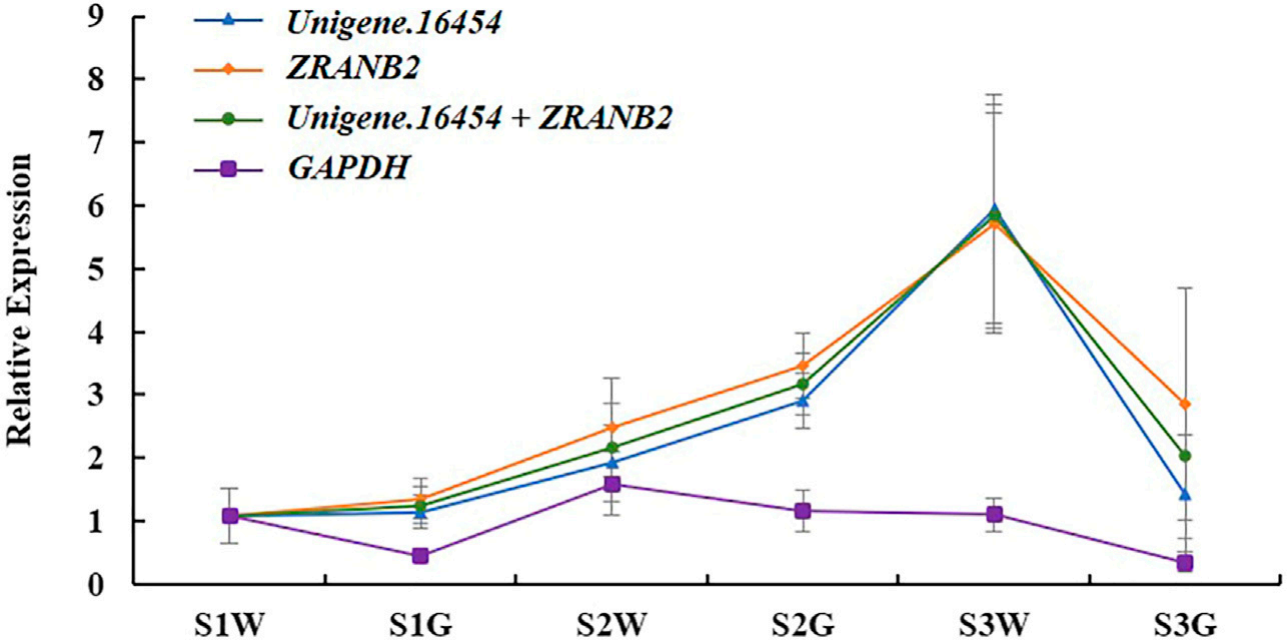
Relative expression patterns of *HEMC*. S1W, S2W, and S3W mean white parts of chimeric leaves at S1, S2, and S3, respectively. S1G, S2G, and S3G mean green parts of chimeric leaves at S1, S2, and S3, respectively. Error bars for RT-qPCR show the standard deviation of three replicates.

In *Ananas comosus* var. *bracteatus* the chlorophyll content of green parts was significantly higher than white parts, which results in leaf albino phenotype (Xue et al., 2019). The *HEMC* gene encodes enzymes for the first rate-limiting step in chlorophyll biosynthesis. Therefore, we chose *HEMC* as the target gene to verify the optimal reference genes in this study. As shown in Figure 5, *HEMC* is expressed in chimeric leaves under all development stages. In detail, the highest expression was in white parts at stage S3, followed by green parts at stage S2. The relative expression levels of *HEMC* were found to be unbiased when *Unigene.16454* and/or *ZRANB2* (Figure 5) were used. However, the expression pattern of *HEMC* changed when the least stable gene, *GAPDH*, was used (Figure 5). These results verified the reliability of the recommended reference gene and provided theoretical support for future insight on gene expression patterns in *Ananas comosus* var. *bracteatus.*

### Expression Profile of *AbGLK1* During Chimeric Leaf Development and Response to Hormone Stimuli

**FIGURE 6:**
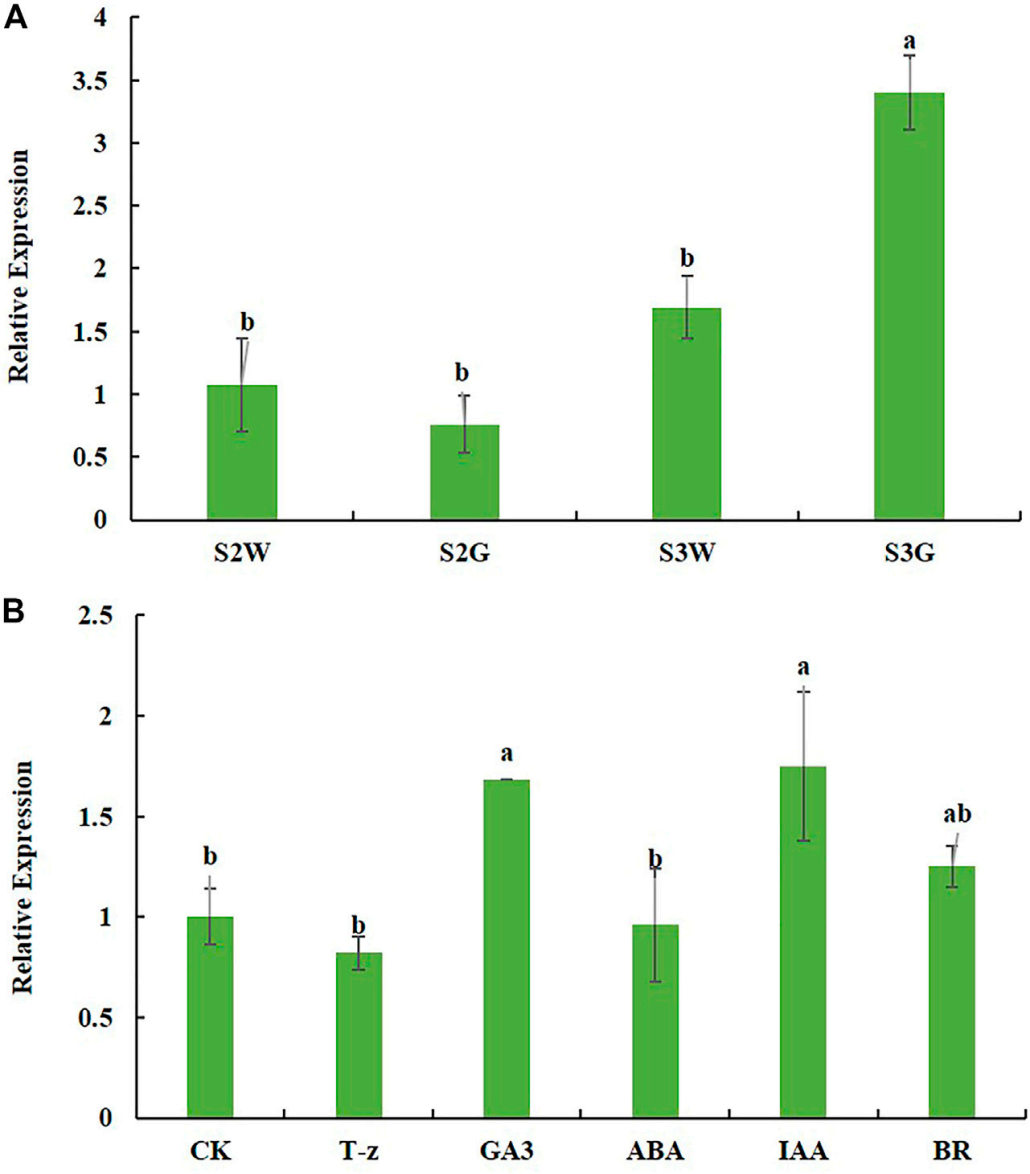
Expression profile of *AbGLK1* during chimeric leaf development **(A)** and response to hormone stimuli **(B)**. S2W and S3W mean white parts of chimeric leaves at S2 and S3, respectively. S2G and S3G mean green parts of chimeric leaves at S2 and S3, respectively. CK, T-z, ABA, IAA, BR mean tissue culture plantlets treated hormone treatments, respectively. Error bars for RT-qPCR show the standard deviation of three replicates. Different lowercase letters indicate significant differences at the 0.05 level.

In *Ananas comosus* var. *bracteatus*, few normal chloroplasts were observed in the white parts of the chimeric leaf (Mao et al., 2020). GLK transcription factors are known to be involved in chloroplast development, therefore, we investigated the expression of *AbGLK1* during chimeric leaf development. As shown in Figure 6A, the transcript abundance of *AbGLK1* in chimeric leaf development stage S2 was relatively lower than that of stage S3, suggesting that the expression level of *AbGLK1* was affected by the stage of development. Furthermore, the expression level of *AbGLK1* in the white parts at stage S3 was about 0.5 times higher than that of stage S2. While the expression level of *AbGLK1* in the green parts at stage S3 was about 3.5 times higher than that of stage S2. These results showed that the expression profile of *AbGLK1* in the white parts differed from the green parts during chimeric leaf development. Therefore, the regulation of *AbGLK1* should be the focus of the investigation. As shown in Figure 6B, the hormone signaling was activated after hormone treatments in *Ananas comosus* var. *bracteatus*. The fluctuating expression level of *AbGLK1* was in response to various hormone stimuli. In detail, IAA and GA3 caused significant upregulation of *AbGLK1* in response to 4 h treatment (*p* < 0.05). BR caused slight upregulation of *AbGLK1* in response to 4 h treatment (*p* > 0.05). T-z and ABA caused slight downregulation of *AbGLK1* in response to 4 h treatment (*p* > 0.05).

## Discussion

*Ananas comosus* var. *bracteatus* is one of the most important pantropical ornamental plants because of its bright pink-to-red colored fruit and white-green chimeric leaves (Ma et al., 2015). Chimeric leaves are optimal materials for the study of plant tissue formation and development as well as the interaction between cells (Burge et al., 2002; Stegemann and Bock, 2009). When breeding ornamental plants, white-green chimeric leaves are a valuable trait for their aesthetic and commercial value. Research into leaf albino mechanisms is beneficial for leaf color artificial regulation of *Ananas comosus* var. *bracteatus.* Gene expression analysis is crucial for obtaining insight into complex molecular mechanisms (Bustin et al., 2005). RT-qPCR is a highly sensitive and specific technique for gene expression analysis, but its accuracy requires appropriate reference genes for data normalization (Bustin, 2002; Gachon et al., 2004; Bustin et al., 2005; Erickson et al., 2009; Kozera and Rapacz, 2013; Takamori et al., 2017). However, reference genes focused on *Ananas comosus* var. *bracteatus* have never been investigated. In this study, we identified reference genes for three specific experimental conditions. Tissue samples were mainly used for gene spatial expression, developmental stage samples for gene temporal expression. And hormone treatment samples are mainly used to study multiple hormone signaling pathways in *Ananas comosus* var. *bracteatus.*

In the present study, transcriptome sequencing data was fully utilized to select 8 novel candidate reference genes in *Ananas comosus* var. *bracteatus*. Compared to novel candidate reference genes, traditional housekeeping genes *18S* and *GAPDH* were identified and fluctuated considerably in *Ananas comosus* var. *bracteatus*. Studies in *Camellia sinensis* and *Brassica napus* have also revealed that the expression of traditional housekeeping genes varies considerably under certain conditions (Hao et al., 2014; Yang et al., 2014). These results suggest that sequencing generates large-scale gene segments to provide a resource for screening superior reference genes (He et al., 2016; Liang et al., 2018; Pombo et al., 2019). In this study, novel stably expressed genes mostly differ from those previously found in other plants. The novel reference genes standing out in the present study are involved in various basic physiological processes, thus providing the basis for their normalization reliability and expression stability. For instance, *ZRANB2* influences the alternative splicing of multiple genes in the transcriptome (Yang et al., 2013). *IDH* is involved in nitrogen metabolism, redox regulation, senescence, and responses to oxidative stress (Leterrier et al., 2016).

Two or more reference genes can avoid misinterpretation for normalization (Xu et al., 2017; Wang et al., 2019). Based on a pairwise variation analysis, in this study, two reference genes were required to present accurate expression patterns in tissue samples, developmental stage samples, and hormone treatment samples. In all samples tested, however, the four reference genes do not necessarily guarantee data accuracy. Similarly, the numbers of reference genes also increased under all tested samples in *Sapium sebiferum* and *Lilium spp.* (Chen et al., 2017; Xu et al., 2017)*.* For the results obtained with all samples set, it should be considered that such samples may be subject to diverse physiological states. Besides the accuracy of data, however, cost-effectiveness should be taken into consideration (Ferradás et al., 2016). These results indicated that it is better to identify reference genes in specific experimental sets rather than simultaneously analyzing them in all tested samples, which was consistent with previous reports.

The use of different algorithms results in potentially contradictory results from the same data. In this study, four popular statistical algorithms (geNorm, NormFinder, BestKeeper, and ΔCt method) were applied to avoid inaccurate results, respectively. Results showed that the stability rank varied with statistical algorithms. For example, geNorm ranked *Unigene.16454* and *Unigene.16459* as the most stable gene under hormone treatments, but the other three programs ranked *IDH* as the most stable. The different results among different programs have also been reported in diverse species, such as *Cichorium intybus*, *Actinidia deliciosa*, *Cannabis Sativa*, *Sorghum bicolor*, and *Vigna sinensis* (Delporte et al., 2015; Petriccione et al., 2015; Mangeot-Peter et al., 2016; Sudhakar et al., 2016; Amorim et al., 2018). However, there is a lack of consensus on which type of statistical algorithms should be selected to rank gene expression stability. To obtain an entirely satisfactory solution, RefFinder was applied in this study. RefFinder calculates the geometric mean (GM) of the ranking values from geNorm, NormFinder, BestKeeper, and the ΔCt method then ranks overall stability (Xie et al., 2004). To verify the reliability of recommended reference genes, the expression patterns of *HEMC* were detected in chimeric leaves at different development stages, using the most and least stable reference genes for normalization. The *HEMC* showed consistent expression profiles when the two most stable genes were used for normalization either alone or in combination. However, the expression pattern of *HEMC* changed when the least stable gene was used. These results further confirm the reliability of the recommended reference gene and demonstrated that the use of inappropriate reference genes may lead to inaccurate results.

Expression analysis of a given gene plays vital a role in further revealing its function. In the present study, the expression level *AbGLK1* was increased with the stage of development. However, the increased rate was inconsistent in the green and white parts of the chimeric leaf. The higher expression level was in green parts at stage S3. A previous study showed that a significant downregulation of *CsGLKs* likely caused the chloroplast defect (Hao et al., 2019). Therefore, the expression silencing of *AbGLK1* should be responsible for the chloroplast developmental defects in the white parts of the chimeric leaf. Consistent with the roles of hormone and *GLK* genes in photosynthetic development, *GLK1* and *GLK2* were both found to be regulated by various hormone signaling in *Arabidopsis thaliana* (Ponomareva, 2012). In this study, hormone IAA, GA3, and BR caused upregulation of *AbGLK1*, while T-z and ABA caused downregulation, suggesting that hormone signaling was activated in *Ananas comosus* var. *bracteatus*. In conclusion, the expression silencing of *AbGLK1* may therefore be due to various hormone signaling pathways in the regulation of *AbGLK1*. The mechanisms of *AbGLK1* regulation should be further verified, which would help reveal its function in chimeric leaf formation.

## Conclusion

The present study focused on *Ananas comosus* var. *bracteatus*, a pantropical ornamental with white-green chimeric leaf. To obtain greater insight into *Ananas comosus* var. *bracteatus*, a pilot study was conducted to identify reference genes to ensure the accurate presentation of transcript levels by RT-qPCR. 10 candidate reference genes were evaluated to identify the appropriate reference genes in three specific experimental conditions, including tissue types, developmental stage samples, and hormone treatment samples. In general, *Unigene.16454* and *Unigene.16459* were the best reference genes for tissue samples, *Unigene.16454* and *ZRANB2* for developmental stages samples, *IDH* and *SDP* for hormone treatments samples, and *IDH*, *PPRC*, *Unigene.16454* and *CCOAOMT* for all samples tested. These results are useful for future research on spatial and temporal regulation of gene expression and multiple hormone regulation pathways in *Ananas comosus* var. *bracteatus*.

## Data Availability

The datasets presented in this study can be found in online repositories. The names of the repository/repositories and accession number(s) can be found in the article/Supplementary Material.
