# Supplementary material for: Validation of Reference Genes for Quantitative Real-Time PCR Normalization in Ananas comosus var. bracteatus During Chimeric Leaf Development and Response to Hormone Stimuli
Source: Front Genet. 2021 Oct 21;12:716137. doi: 10.3389/fgene.2021.716137 (PMC8566434; doi:10.3389/fgene.2021.716137)
Supplement: Supplementary file 1 [file DataSheet1.ZIP › Addition files/Additional File 1.docx]

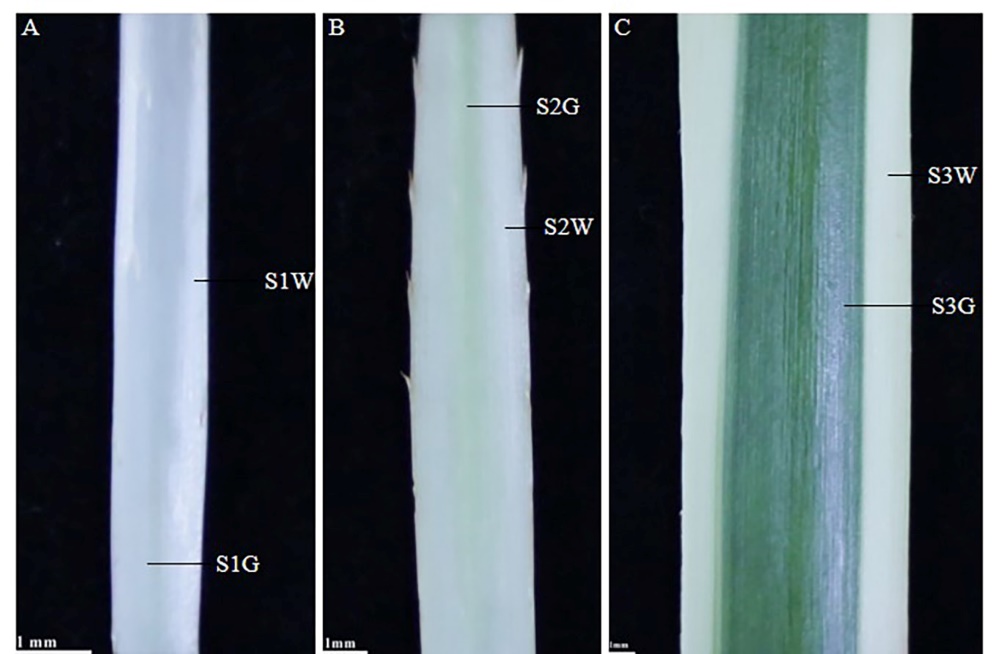


Fig. S1: Illustration of plant materials applied to the experiment. (A) chimeric leaves at stage 1 (S1, no chlorophyll is visible on leaf), (B) chimeric leaves at stage 2 (S2, chlorophyll becomes visible on leaf center), (C) chimeric leaves at stage 3 (S3, two thirds of leaf are fully pigmented by chlorophyll). S1W, S2W and S3W mean white parts of chimeric leaves at S1, S2 and S3, respectively. S1G, S2G and S3G mean white parts of chimeric leaves at S1, S2 and S3, respectively. Bar = 1 mm.
