# Supplementary material for: Validation of Reference Genes for Quantitative Real-Time PCR Normalization in Ananas comosus var. bracteatus During Chimeric Leaf Development and Response to Hormone Stimuli
Source: Front Genet. 2021 Oct 21;12:716137. doi: 10.3389/fgene.2021.716137 (PMC8566434; doi:10.3389/fgene.2021.716137)
Supplement: Supplementary file 1 [file DataSheet1.ZIP › Addition files/Additional File 2.docx]

Table S1: cDNA sequences of 8 novel reference genes

*Unigene.16454*

ATAATTGATACTTGTTGGACTTGGATTTGACTTAGGAAAAAATCCCGAATTGATCGTCATTTACATACACTGCCGGATTTAGCTCTAGGAGCCGTGTTGCATTGTATCGTCGCGGGCTTTGTGCGATAGATATCCAGGGTAGTGTAGCTTGTGTTTGTGCTCGTGCTGGGACGCCGAGCTTATACTAGCTGAGTATAGACTGTTACGGGGTGTCGGGCGCCGTCGGGGCAGGCGGTGGGCCCCAAAGTCTGTTTTGGTTACTTGGGTTTCGCTGGTTAGGACCTGATTGAGCTCGACAGAGCTACGCTGCATACTCGGCTGGGTAGCTCCCCCGAGTGCCACTCCGGAGTTAGGCTTTGTGCTTTCGCGTCGGTGTCGGGTAGTGCAGGTTGGACTTGCTCTCCACTGAGGTTTAGAGTGGGGGTAGCTGTGCAGTCACAACCGGGCACGGGACGGGTGCGTTCACAGTCCCAGGGACGGGTGCGTTCACAGTCCCCTTCAGATTGGGTCTGGTTGACTTGAGTCTATAGGTGTTAGTTCTGGGCATGATAGCATAGCGACTTGTAGCTGTAGAGAGTTTCCAGCTTCATTTTCTTCTATCTTGCTTACCCTGTAGACTTAGTGGGCGGACCGATGTTGCTTAGGGCGGCACCCACTGAGGACTACTTATTTTTATAGTAGTTCTCACGCCCTCTTTCTTCCACCGATGTTGCAGGGCCT

TCTGTTTCGGCTGCTGCTCCTTCTGAGGCGGACCGCGGGAAAGGCGTGGCGAGTTAGAGCCTACCCTTCGAGTACAGTGCTAGAGGACTCCCGCTGCAGCT

*CCOAOMT*

AATAGAGACGGCGGCAGAGAGTAAGCCCATCCGCGATCGAAAGCTGGGATAGTTCAATGCGGGGGTCAGCGGCTAGCGCCCTGTTAAGATTTATCATGGTCTCCGTATACACCAGAGCTTCTGGAGGCAGAGGCTCGTCCTCTGGCGAAGGTGCCACCGATCCGCACCACAGGGTATTGTCGTACGCGACGACTCCTCCCACCCTCACCAGCTGCAGAATCCTCTCATGGTAATGAATGTAGTTTTCTTTATCGGCGTCCACAAACACAAAATCAAATGTCCCCTTGTTCTCCTCC

*SDP*

GTTTGGTTGCGTAGCGTAAATCGCTTCCTTTTATTCCCTTTATTATCCCCTTTATTACGCACCCAATTCGGATCCCTCGTTGTCTCTTTCCCCCAAAAAGCAACCATTTTTTCCCGATCGATCGATCATAAGCTGCGATACTCAGATTTTGGTAACTCAATTTCTCTCCCACGGTTCAAATTTTCAATATTTTTATATTGATTCTACAGCGCAATTTGCGGTTCAAGCAAGGGAACGAGGGGGAAAGGATCGATTTCTTTTGGGTCACACTCTACTCAATTCTCCGCAATGTTTCTCGATTAAATTCCCTTCAAAGACCCAAGTTTTCCTGATCAGAGAGACTAATTAGATCGAATTCTCCTCCAAAGGCACAAACTTTATCGATCGGAGAGCTCTAATTAATTCGCTACGAGATAGCCGAGAAAGAGAGAGAGAGAGAGAGAGAGAGAGAGAGAGAGAGCACGAGATCGCGTTGTAGTTTGAAATGGATATCACGAGCGAGGCGTCGGTGGATCCGTTCTCGATCGGGCCCTCGACGCTGCTGGGGCGCACGATCGCGTTCCGCGTCCTCGCGTGCGCCTCCCTCGCCCACCTCCGGCGCGACGCCGCGCGGTCCGTGTACGCGGCGCTCCGCGCGCTCCGCTCCGCCGCGCTCTGGTTCCACCCCCGCAACACGCAGGGGATCCTCGCCGTGGTCACCCTCATCGCCTTCCTCCTCCGGCGCTTCACGAGCGTGCGCACGCGCGCCGAGTCCGCGTACCGGCGCACGTTCTGGCGCAACATGATGCGGAGCGCGCTCACCTACGAGGAGTGGTCGCATGCGGCCAAGATGCTCGACCGCGAGACCCCCCACAAGGCGAGCGAGGGCGAGCTCTACGACGAGGAGCTCGTCAGGAACAAGCTCGACGAGCTGCGCCGCCGCAGGGAGGAGGGATCGCTCCGGGACATTGTCTTTTGTATGCGCGCTGATCTCCTGAGGAATTTGGGGAACATGTGCAATCCCGAGCTCCACAAAGGAAG

GCTCCAGGTGCCTAAACTCATAAAAGAGTACATCGATGAGGTCTCGACCCAACTAAAAATGGTATGCAACTCTGAATCCGATGAGCTACTGTTAGAAGAGAAGCTTGCGTTCATGCATGAGACAAGGCATGCCTTCGGCAGGACGGCCCTTCTCTTAAGTGGGGGCGCTTCCTTAGGAGCCTTCCACGTGGGTGTAGTGAAAACACTAGTAGAGCACAAGCTCCTTCCCCGTGTAATCGCAGGATCGAGTGTCGGTTCAATAATGTGCGCCATTGTAGCGACCCGGTCGTGGCCCGAGCTCGAATCGTTCTTTGAAGATTCGTGGCATTCCTTGCAATTCTTCGACCAATTGGGCGGCGTATTTACTGTGGTCAAAAGGGTAATGACATGCGGAGCGGTACACGAGATCAGGCACTTGCAAAAAATGTTAAGGCACCTCACGAGCAATTTAACTTTTCAAGAAGCTTACGACTTGACTGGTCGGATTCTCGGCATCACGGTTTGCTCTCCCAGAAAGCATGAGCCACCACGATGCCTTAATTACCTGACTTCTCCGCACGTCGTCATATGGAGTGCCGTCACGGCTTCTTGTGCTTTTCCCGGGCTCTTTGAGGCTCAGGAGCTGATGGCGAAGGATAGGTTTGGCGAGATTGTTCCTTTTCACGCGCCGTTCTTGGTGGGTGAAGAGCAGTCATCGCAGGGGACCTCAGCACGAAGATGGAGGGATGGAAGCTTGGAGAGCGATTTGCCTATGATACAACTGAAAGAATTATTCAATGTGAATCACTTTATCGTTAGCCAAGCCAATCCACACATTGCCCCTCTTTTGAGGTTAAAGGAGCTTGTTAGAGCTTATGGGGGCCGCTTTGCTGCAAAGCTTGCTCAACTCACCGAGATGGAGGTTAAGCATAGGTGCAATCAGATCCTGGAACTCGGATTCCCACTGGGTGGAATAGCAAAATTATTTGCTCAGGATTGGGAGGGTGATGTCACAGTGGTCATGCCTGCTACGCTTGCTCAGTACTCAAAGATCATACAAAACCCATCTTACGCCGAGCTCCAAAAGGCCGCAAACCAAGGCAGGAGGTGCACTTGGCAGAAGCTCTCTGCGATTAAGGCAAACTGTGCTATCGAGCTCGCGTTAGATGAATGTGTCGCACTTCTTAACCACATGAGGAGGCTAAAGAGAAGCGCGGAGAGAGCAGCTGCCTCTTCCCAAGGACAACACACGAGCGCCTTTCAAAGAAACACTTCGCGGAGAATCCCTTCGTGGAATTGCATAGCGCGGGAGAACTCATCGGGGTCTCTGGAGGAAGATATCATGTTGGAGGCTGCAAATACTTCAGCCCAACAAGGCCCAAGTTCATATACTCGTGGACCAAGAAGCGTACATGATGGAAGCGATAGTGAGTCAGAGAGTGCGGACTTGCATTCTTGGACTAGAAGTGGTGGGCCGTTGATGAGGACTGCCTCCGCAAATAGATTCATTAACTTCGTTCAAAATCTCGAGATTGAGTTGGAATTCAATCAGAATTTTACGAGAGATGATGAGAATAGAGATCCTTTTTACAGTAATTCAAGAGTTACTACTCCTGATAGAAGCTCAGAAAATACAGACACCGAAATCGGAAATTGTAGAGCTCGTCTCGGAGCTCCTAATAGGATTGTGATTTCCGAAGGAGATTTGTTGCAGCCCGAAAGGATGCAAAATGGAATTTTGTTCAATGTTGTCAGAAGGGAGGCTTTACTTGGGAATAGGGGCAGCGACTTCGAGCAACAGCAGCAAACTGCTTCAGCGGAAGCTGATATAGAGAGTGCACAGGTAGAATCCTGTGATGCTGTTTCAGCTTCCGATTTTATTGAAGATGATGAAGAAGAGACCGAATTGAACTGCAGTGGCCAATTGGCTTCCCGCACTGATGATAGAAATTTGGATCAATCTTTAGCGGATCAAGCTAATGTCCATGGGCTAGCTGATGGTTGAAAACTAATTTTAATCTTTTCAGAGAGAGAGAGGACTAAAATGAGGCTATGAAATTGAAGCAGAATTAGCATCGAGTTGAGCTAGGATACTTCTAAAAGTATTACTATCCACATACTTTTAACTTTTTCACCCTTAAATCTACTACTATTTCACCTGCCCTCACTAAACCTCAGGCATCCAAGAGCTACAGAGGGTGGAAGCAAGTGAGTAGTAAAATTTTAGAAGTATTCTAAACACAATTTGATATTCGAGCCGGGATTAGATTACTTTGCAATCAGATCCGAGACTTGAGAAGTTCTTCTCAATGTATATAAGCTGTTTGAATTGTGGATTTATAAAGGTGGAGGCCGAAATGAGAGCTGATCAAGGTCACATGTAACTTTATAACATGTTTTAAATTTTCCTTTTTTTCATGG

AAAATTAGCTCTATAGCAAATTGTAGGATACACTATTGAATATTTGTCTACAAAGCTCCTGCATAAAGCACTCGCAGTTGTCGTACGTGATGTTGAGAATGTAACAAATATAATCCTCAGAAAGTAATTTGATCTTTATGTGGTTTTTTTCTTCATTGGTTTTAGTATCTAAC

*Unigene.16459*

ATCAAAACGGATTTGAAGCATCATGGCACATAATCGAAGACCTTTAATTTTCGGATCATGAATTGGAGGCCGTTCGGGTGGTCGCGCGAGAGATCGGGCCAAACCGGGTGCCGGGTGCCGCGGGAGGCCGATTGCAAGTGTCGACAAGCAATCAGAATGCGAAATGAGGTGGGTTGTGCTCACCGAAGCAACTAGGTCGCCTATATGTCTAAGAGTAATGGTTTTCTAATTGATGCATATAGTTGTGTTGGTAGGCTTACATAATGATATGAATGCTTGAAACATACTATGTAGTTGAGATTACCTATCAATGAGATGAGATGCATAATGAATTAAATGGTAAAAGAATGCATGAAATGCTAAGTATGAACTATTTTGGATAATGACTATATGTATGTTGACATATCATGTGATGCTAGTGGTATATGTGCATGATGACATATTATGAATTAACATATTGTGGATTATGCTAAATAGAAAAACATACTTGATGACATATTGCCATATTGTGGAACATAGTAGATGGAAATGTATGCATGTTGATATACCTTGTGTAAGATATAGATCCAGAGGATCTAAAAGGTAAGTAACACCTAGAGGGGGGTGAATAGGTGTAAAAACGGCAA

*ZRANB2*

TGATGGATCTGAAAAACGTTGAATCAAATTGAAAAGTGACAAAATACTTCGCTTGAGATCCGCACAGATAGGTGCGAGCGCACGGTTGACGAAATTACGCCTCCATTAATCAAAGCTGTGGTAGAGCGCGAGCGCGTGAGAGAGATGCGACCTCGCGAGGACGAAGAGGCGGCGCCGCCGCAGCCGGAGTCCTCAGACTCTCCGGCGCCGCCCGCGAGGCCCGGCGGCGGACTCAGCAGCATGGTGGTGCGCCCATCGGAGAGCGGCGGAGACGGCGGCGCCGACGAGCCCGGGGCCGCC

TCCCTCGCCGCCTCATCACCGCCGTGACTCGCCCCAGGGCGCGCGCCGCCGGAGACCCTGGCCGTCGCTCCGCCGCCGCGAGTCTCCGCCGCGGCTCCACCACCGCCGGGGGAGCCCCCCGGATCTGCGCCGGCGCGGGAGCCCTCCCGGTTTGCACCCTCGTTACGATCGGTTCCACGAGAGCCAAGGTCTGCGTTTCCCTGAAACCCTAGATTCGAGATGATCAAATTCTTTTCTGAATCTGATTAGGATCTTTTCAATTTTCTAGGATTTTATTGTTGAATAATCTCAGGCGCATTCTAGTTTCATAGTGTTTAGCATATTGACATTTTCGTTTACTCCCTTTTTATTTTTGACCTA

TCTGGATGAGGAAGCTTGCTTGTTCCTCTCTAATTGCGCATTTACGCTGGTCGAACGTTGCTCCTATGGCTATTTATGGTCTAAATCGCTCTACATGGTTATGGTTGCGTTCGCCAATGTAGGTGATAATTGTTGTTCCTTGCTGAATGATGGATTAGGTTGTTTCTTCTCCACCTAGTCATATAAAATTTTGGATCCACTAGTTCTCTAATGATCTAAATATTTTATTTTGCAATTAGAAATTTCAATATTATTGTCTAGGGCCTTCTGTGATGCACTGTTGCCTTGGTAAAGTAATGTAACACTACTTTAACACGCATGATTCAAGGTTGGAGGAGATGTTAATAGATGTTGTTGTTATGACCACACTATTTATATTATTTGATTGAGCTTGTTGGATATTACTAGGTACGGAGTGAG

GAGAGATAGGAAAACCCTTTTATTGCCTTTTCTCTCCTTCAGTTTCCTTTGCTGCTTTGACTGTTTATTTCCTTTTTCTATCCATAGACAATCTCCTAAGTTCTTATTTTTGTATTCATTGCCATTTCAAATCGCATGCCAGAACTTTTTTAGTAGAAAGTATTGCTCATGCTTTGCTGTACCTGATAAGATATTAATAAACCCTTGGACAAACCTGAACAAGCCTGCTTTTAGCCCTACCTGACTCCCTTAGCTATGCTTACTTAACAAGAAAATCAATATGCCAATTAAGTTATGGAATATTTCAGAATCATTCTTCTCCATGCTAGTATTAATTTAGTTCGTTTGAGGTATTGTTAAATTTTGTCAGGTGACTGTAAATTCTCATTCCAGTATTAGTTTTGGTCCTTTTAAGGCATTTCTAAGTTTGCTATGTAATATTCCTGAGCTTCAATAGGAACTATTTAACTCTTGTACACTGTAATCTATACTATATTTTGTTGTTTCAGCCTTTACGCTTTTTGGAGCAGACAGTTGAGAGGACGTCATAAAAATTAAGTAGTTGTACTTAAGGATCACTACTAACTAGTATGAACACTTTTAAATCAGTTCTATTGTATTCTCTTCTAACTCATCATTTCGCTTCCTTATCATGTATTCGTACTTTTAATGTTCGTAATAAATATCTCTTTCCTTCTTAAACACTTTAGATATAAGGTGGGTTATCCACAGTGCAATGACTATATTCTGGAAAAAAAAAAAAAACGTATGATGAGCTTGATAATCCTCACGTTGTTTCTTGTTTTGAGCACCTTTGGCTAATTCGTTTGCTGACTTATGTTCAGTGTAATGATCAAGCACTACAAGCTACCCTATGTGCCAAATGGGGAACAAAATTTGCTGTTCTGCTGTAACAGTTTCCAAGTTGGCTGGCTTGGGTGCAAGATCATTATTAAGAAGACTGTTTAGTTTTTTTTACTCAGGAAATTGCTTGATGGAAAAGGTTTTGGGTGATGAAGGAAAAGTGTGATGGATGTAAGAGACTTAAAGTGGATGCCTTGAGTTGTCCTCATGTTTATCAGTTGTTTGCTTGAAGAAAGAGGAGGGCATGAAGAGAGAGTGTTTGTTCCTTTTTACAGAGCTTTATGGTTCTACGCTAAAATTGTCTAAATATATGCCTTACTGGTTTCTAGCGCAAATAAACTTAGTATGAGCTCATTTACGTTCTTAGATGGGGTTATCTGGAACGTTTTTTTGGGTTCAATATCCAAATATTTTTGGGACTATCAGGTTTGTTGCTGAGTATTATCAACTCAAATGGGGCTCGTATTCTTTTTACTGTATTCAAGTATTGCAGTCAAAGTCGGCATTATTTCCTGCGCATAGGCTGATGACTAACCCTAACGGGTGAAAATATGGTTACTTGGGTAGCTATCGTATAGCGTGACAAGTAAATTTTCCTGATGTTTCGGAATAAGGGAAGCAGACAAAGGCTGAAAAACTTTCTGTATACAAAACCATCATATTGGTAATGCTGTTTGATATGTTCTCAATGTGGTTGAGTAATCAGTGACAGGATCAGTTATTGTTCTCAATTGGAGAAGGGCATTCTGGCACGTA

TGACTTTTTTTTCCAGCTCAACTGGATGGATAAGCACTTCTGAACCTTCTGCAGTCTAAAGCATTCAGACGCCAAAGAAATATATTGGTTTTATAAGTTATCGCCTTGGTAGATCCTTTTGGCAGTTTGCCTGTTCGCTCACATCCATATCCAGCATGTCATGTTTGGTAGAATGTGGATCTGCTGAAATGTTTATGGGTCTGCAAGGGATTAAACCTCTATGCTTTTGAAAATGAGCTAACTGTTGTTGGAACTAAACTTGATGGATGAGAACAGATTGGTCATGGAGATGGAAGATTTTAGACATTTACTTTTTGGATATTTAAATTTTGGCTTTATGCTACTTCAATTTGGTGGAAATTTCAATTTGGTTGTCAATTTTGAGAGTGGCTGGATTGGTGCTATATGATTTAACTCTTCTTGGGGAATGCCTGCCATTTACTTAGTATTAAAGCTGTGAAGCATGTCGAAAGCTGGCTCATTTACAGTATTATCAAAAGCAAGACGGTATGAATAAGTGAATGGGGCTACGAGAACTAATGTAGCTTTTAGATTGAAATCTTTCAATGTTTAGGTCTAAGCTACATGAAGCACCATATCGAGACTGGTGAAAGATACTGTTTTAATGTTGCACCTCATCTAGTTTTAAGGACTTGCTACTTATTCACATTAACTACTCCTTTTCTGCATTGAGGAAAGCTTTTGTTGCAGCCCTGCAGGTAGTTCTTTTTGAAGTTCTTATCTGCTATATCTGCCTGATGGAAACAGATCATCCCTGAGTGACTATTTCTAAGTTTAACATCAAGCCTTGTATAAGTCTTTTATTGGTTTTGTTTCCAGGCTATGCTATGGGTGCAGGTTCTATTTCACCTCCACGGCGAGGAAGATTTGATGATGCTCAATATGGGCCAGATTATGATGACCCAGTTGGTCCACGCTATGTGCGTGGATTCAATAGTGGAAGAGGTGGCGGCAGGTTCCGGGATGTTTCACCAAATTATGGCTTCGGAAGGGGCGGCAGGTCATCTGGTAGAGGTTATGCTGGAAGGGGTTTACAACCCTCTGAAGGGGAGTATGTCCACAGAAATGACCCAAATTTATCGCCTAGAGAAGGTGATTGGATCTGCCAGAACCCGACTTGCGGAAATCTGAATTTCGCCCGACGAACTCACTGTAACAACTGCAACAAGTACCGCTATGGGCCGGAGCTCTGCAGGTCGAGTCGCAGTCCCCGGAGAGGCTACACTAACTCTCCTCCTCCTCACGGGCCTCTACCTCGGGCTTTTGTCCCACCACTCATCGAACGCGACCCACGTAGGGGTATGGAAAGATACAGATCCCCAGCACGAGGTTGGGCGATAGATGATCCTCGAGACTTTCCAGCTCGGCAAAGGGAAAGGCTGTACTACCGTGAAGAGCTCGACTATAATAGGGACCGGGCAAGTTTCGACTGGTCGGTATCCGACGAGTGGGATCGTGCACGGGACCGCGGACGTGAATCACGGGATCAGTTTCTGACCGATAGAAGAGGATATGACCAACGGTCACCGTCGCCGCGTGGGCATTGGGGACGTAATCTGAGGGGGGAGAGGAGCCGCTCGCCGACAGGAGACAGAGCATTGAAAGGTTCTTTCATTGGGCGGGGTCGAGATGATCGAGACTATGCTGATTCATACGTGAGTCGGGCACGAGCTCATCATTTGGATGGCGGCCCTGGTCGCAGTGGCTACAGGCAGGGGAGTGATCCTTTCCCAGGCCAAGGACGAGGTGAGCGGCGGGCCATGGGCCGTGGTCGGAACGCCGACAATTACTAGTGGTTGACGAACCTCATGGGATCAAATGCTTTGAGTGAAATCTTTAAGAATTATTTGGCTAAAACTGCTTAAAAGTCTCTGTTGTTGAGAACAGCTGCTTTTTGTTCCTGACTTTGCCATTGATGGGTTTTTGGATGGGACTAAGCAGCTACTTTGCCTCTTCGGAGACCTCCGATAAAACTAGGCAGAAATTTCATATCCATTTTGCTTTAAGTGGTTTCTGTATTCCCTTTCATCTTACATCTACCATTAGATTCGAACTCTATGCTGCTTTTCGGGGATATCCTTCACTCTAAAATATCATGTCCATTTTGGCTTCATTCTAAAAATACTATTCTTGAACCTC

AATTTGGACAGATTTTATTTCCGCCCTTACAGTTTTAATTGTACTTATTTAGTACCTGCAGTTTCTATATATTTTTAAGGTCACCCTTTGAAGCTAAAAAAAGAAAATGTCATGACGCTACAATCATTCAACTTTTCTTTGGCGAGATTGGAACTTCATAATAAAATTGTAAGCGAAGTTAAACTTTCACAGAGCTCAGAGGTAAAAGCTGAAAGCAAGGTTGCATGTTAAGAAATACACTGAAAGTCCGAGGTTCAAATCAAAATTTGGTCAAGCTTTAAGAATTAAATTGTTCATAACCATAAATTAAAATTTCGGCTAAGTTTTAGGGACAATATAAAATTAACCTAAATTAGACAC

TACATACAAAAGAAGAAAAATTTACGGACTAAAACTCAATTTTGCCAAAGTTCAGGGATTCCAAAGTCTTAGAAAGAAAAAGGAAAGAAAATAAGAGAGTTTTTCCCTCGCCTCTTAAACCTCATCTCTTCTTTACATCGTAGCTATCGCCTCTCACACCACACCACACCACACCACACCACAATCCACTTCGTAGCTCTTCCTCCCATGGCGAGCGTCGTGAGCTTCGCCTGTGCGAAACCCATCGTTTCCCCGACCCCCAAATCGTCCCACGCTCTCCCCACCGAAGCCCCCAAATTCCATCTACTCTCCCACCCGAACCCTAATTTCCATTCCCTCAAGGCCAAGGCTACGGAGAGCAACCAGAGTACGAAAAAGACTAGCATCGTCTGTATCTGCTGTGAAGGAAATGGTGCGGTATTATGCACACAATGCAAAGGAACGGGAGTGAATTCGGTCGATCACTTTAATGGTCAATTTAAAGCTGGTGCTTCATGCTGGCTTTGCAGGGGTAAACGGGAAATCTTATGCGGGAACTGCAATGGGGCAGGATTTATGGGTGGTTTTCTGAGTACTTTTGATGAAACAGCTCAATAGTGCTGAACACATACTACGGCCATGTAGTTCGTGCTGGTTTTAATCTATTGTATCTGCAATGATTTTCGTGGAAGGATAGTTTCCTTCTTTAACAATTACTATAGGACTTGTATGAGACTTTTC

TTTTACGAAGATGTGAATTAATTTTAGTTATTATTTGTGGAACCAATGGATATTTGTGGAACCAATTGATACTTGTGGACTTATCGATGGTACTCGCAAGCAGATTAATAGCGTTGGAATTTGAGGACAAGAAGTTGCATGTTGCCTATGTTAAATA

*PPRC*

CTCGACCTAGACAATGTCGTGCTGCGCTAGATCGCACCAAGCAACGAGCCTATCTATTTTTCTACATTTTTGTTTTTTTATTTTAAAAATTTTAATTTTATAAAAACATACTTATTTATTAAATAAAAAAAATAAATTTAATATTTAATATTTTTCCATTGCTCGGCCAATTACTCGGTGCTTCCCGAATCCCAAGTAGTTGCAGGTTTCATGGGCTTATTTGGCGATCGTAAAAATAAAAAATAAAAATAGTAAGATTTTTAATAATTTGTATTGAGGGTTTGCGAAACAGGAGCTTATAGTAAGCCCGATTTCCCTATTCGGCGGGCACTTCCCCTCGCATTCTTCTTCACCCAAAAACCCTAACACTCGCTCCGAAGGAGAAAAGAAGAAGAGGGAAGTTTGCACAAGGAGAGGAAAGCTTATATGCAGGCAACGGCCGCTTGGAAGATTTTCTTCTCGATGCAGGCGAGGGCTGCTTGGAGACGCCTCTCCCACGCGCCTAAAGATTGGAACTTCCAGAGCCCCTGGATCTCGCCTCTGCTTCCCCGCCCACTTCTTCCTGCTCGTGTACCGCCCATAGTCGAGTTCAGCACGACCCCCAGCAGGCCCATGAGAGGCCGCGGGAGAAGAGCCGGAAGAGACGACGGCCCTTCGGAAGAGGAAGAGGAGGAGGATTTCTTCCTCCGCACTCTCAACTTCGGCGACGACGGCGGGAACGAGAGAGCAAGAGAGAAGAACCTGGCCGACCCCCAGCAAGGCCCTCCGAGGCCCGCTAGGAGGCCACCGAGGGGCGAGCGAAGGTCGGACACGCCGCGCGATGTCGAGGCAGATGATTTCTTTCCCCATTTCCAGGATGGAAACGAGATACTGCTAGGCGGCGGCCGGAGGTCATCTTCCTACCGAATGCCCAATAGACCGCCGCCGGCAGGAGAGCGAAGGGGGAAGGCTCCTGGAACTCTCCGTCATAATCTGAAGGCCGGTGACATCGATGAAGATGTATACGGAGATTTCGAAGCT

TTACTTCGAGAAGAAGAATCTCCTCCTCCTCCTCCTCCTCCTCCGAGACCCCCCATGAGACCCAAGGAAGCTGGAAAGAGTGATGGAGCGATCAAGATCAACGACGAGTCAGACGATCCCTCTACCAGGGAGAAAGGCGGTGCCGGACTAGGTGAAACCCTCTTTCAAAAATTGAAACTTGGGGATGCTGCTCCCGGCGATAAAATGGAAGGTGAAACTCAACGCAAGTCCCCTGCAAATCTCTCCGACACCGATTCTGCAGCAACCGAGCCTCCGCCCCAAGATGCGGAGGAGGTCTTC

AAGAAGATGAAGGAGACGGGCCTTATCCCAAATGCGGTTGCCATGCTTGACGGCCTCTGCAAAGATGGGCTGATTCAAGAAGCCATGAAGCTCTTTGGGCTGATGCGTGAGAAGGGAACCATCCCCGAGGTCGTCATCTACACTGCTGTCGTGGACGGCTTCTGCAAGGCCGCTAAGTTTGACGACGCCAAGAGGATCTTCCGGAAGATGCAAAAGAACGGGATTGTACCCAATGCTTTCAGCTATGCAGTTCTGATACAGGGCTTGTCCAACGGTGGGAAATTGGATGAGTCTGTTGAGTTCTGCACGGAAATGTTCGAAGCAGGGCATTCTCCGAATGCAACTACCTTCATCGGGTTGGTCAATGGGTTCTGTAAAGAGAAGGGGGTGGAGGAAGCCGAGAAACTTGTAAAAAGTTTTCGTGAGAGGAACTTCATTGTTGATGAGAAGGCTGTAAGGGAGCATTTGGACAAGAAGGGCCCCTTCTCACCGCTAGTTTGGGAGGCTATCTTTGGTAAGAAGATCTCGCAGAGGCCATTCTGATCTCTTCTTGTTCTTGGGGTTGCTTGGAAACACGCAATGGGAACCCATGACTAAAGAATTGAGTTTGGGAGAATATAAAACTTAATCTGGCGTAAATGTGAATATATACTCTGTATT

GCAACCTTCGCAAATCAAGAATATCCGATTAAGGTGAATCTTTCTGAGACAAGGAAGGCGAGCACTCGGTAGTCCTTGTGGGTTGAACGCCTGCACACAAGTATTAAAGAAAAACATGATCATATTTCATTAGCTGTTGCTTTATGGCTTCCCTATAAATTGCCAAAGCATCGCTACTTGCTGGGTAGTCCAATCCCAAGTGATGTCTTATCTTATCTAGACATTTATCTTTTGAAGTAATATCTGGATTAAATAGAAGTTTGTCATTCATAGTCGGTCGATTAAATTTCTGGACTAAATCGGTAAGCAATAATATGTTATCTCGGTGAGAAGCGTAGAACTGGCACATGTTTCGTATTGCATAGTATT

*IDH*

GCTTGTTTGATAATTTAATGTTTGTTTATATTGATAATCTTATATTGGCTCTTCTACTTAATTGATAAAATCAGTCCCAGATGGAGATGTGCCTGTGGAGCTAAATGTTTATGACTTCCAAGGGCCTGGTATTGCATTAGCTATGTACAACGTAGATGAGTCTATTCGGGCCTTTGCTGAATCATCAATGGCAATGGCACTTGCTAAAAGTTGGCCTCTATACTTGAGCACCAAAAATACAATTCTGAAAAAATATGATGGCAGGTTTAAAGACATCTTTCAGGAGGTATATGAAGAGAAATGGAAGGAAAAGTTTGAGAAGCAGTCGATATGGTATGAGCATCGGTTGATCGATGACATGGTGGCCTATGCATTAAAAAGTGAGGGAGGGTATGTCTGGGCCTGTAAGAATTATGATGGAGATGTTCAGAGCGATTTTCTTGCCCAAGGTTTTGGTTCATTGGGCTTAATGACATCCGTACTGTTATCTTCAGATGGAAAAACATTAGAAGCTGAAGCAGCTCATGGTACTGTTACTAGACATTTCAGGCTACACCAAAAGGGACAAGAGACCAGCACCAATAGTATGGCTTCCATCTTTGCATGGACTCGATCGCGGTCTTGCACACAGGCACGTTTTGTTGTTCATTTGGAGATAGCTTTACTTCTTGGATTATGTGATTTTACTATTTGTGAGTTGGATTTGGTACATCATATAACAATGTTTTTGCCTAGTTCTCACTTACACTGCATGGTCTTTTTTT

*Unigene.26260*

CATCCTAACGTAAGAAAAGTGGATAGCTGCCATGGTTCAGACTGCAAGGAAGTTGTAAAACATAGAATGGTGGAGATGTAAGTAAGACACTGCCGAGAAGTAAGGTGGGAGGTGGAGGAAGAAGGAAAAAAAGAAAGAAGAAACATACAACTAACAATGGAACCAGATCAAGAGAAGAAGTAAAGCAGCAACATCAATTAGAGTAAAGAACACCACAGCAAGTGAAGACAAATATGTAAAGCAAAACAAAATAGGCTAAAAAACATGCAAGGTCAGTACACAGACAATCTGGAAAGACAATATAGAAGAAGGGAAATCGCTGAGGCTCAGGTTTAAAAGCAAGGGCGTGGCATCTCTTTTTTTTTTGCAGCATAAGTTTACAGCAGGTGGCAGAATCTAACGAAGCCCACGGTAGTAAGCAACAACAGGAGCTGCATTAGTAGGTGGTTGCCTTTAAGTAGGCCATCCAACCTAGAGTGAACAGCCGACCAATTAATACCAATTACACCCTCGAAATCACACCATGTATATTGATGGATGGTTGCGCATGGGAAAGCCATACTGCATAGTGTAAACAGTTGCTTTCTAATGCGCCAAACCACTACACGCTTCCTTTTTTTTTGGAAAGGCATGGCAGGGAGTAAGATAGCCGCAAGGTGTAAATGTAAGAGATAGTCATCATTGCCTCGTAAAGAAAATAAAAAAAAGAAAGAACCAACAAATCAGGATCAGCACAAAAAAGGAAACCAAGGAAATCAAGAAGAAGAGTATTGAAAGCAAAATAATAAAGCAAAGAGAAGAAAACAGAGAAGTAAGAAACACAGGTTAGAAAGAAGAACAAAAGAATAAAACAGACTATAAATGAGCACGAACATAAAGGAAGTAGTTGGAACACGCAACCAATCAAATAAAACAGGTATGTTCATGTAAAGTGTGAGAAATGGAACCGAGCAAAAGCATTCCCGTCGTCGGTTGCACAGGCAAATCTTGGCCGAACTTCATGCTGGGAGAGAACACGGGGTCAGAAAAACAGGGGGGGCAGAGCCCAATATAGTAACATCAGTCAATAAACAGTATTAGATGGCTTCCCTGTGATAGGCCGTCCAATCGCTGTTAGATGGTTCCACAGTAAAAAATATATCTCCCGAAACAGCAGCCAGTCCCAGTGTAAAAAAAGTTCAGTTCTTTTTTTTTTTTGTGTTACAGTCGTCATCTATGCACAGCAGCCAGAAACAGCAGCCAGTCCCACAGTGTAAAAAA

AGTTCAGTTCTTTTTTTTTTTTGTGTTACAGTCGTCATCTATGCACAGCAGCCAATCCCAAAGTGTAAAGAAAGTTCATTCTTTTTTTTTTTGTGTTACAGTCGTCATCTATGCCTCCCAAAAAAAAAGAAAAAAAATAAACCAAAGGGATTAGAAGAAAAAGCTAACAAATCAAGAGCATGGACAGTAGAAAACAGGAACACAAGAAACCAAGACACAAGAAAACACCAATATAGCAGTGCGAGGCAATAGGTAGTAGTAGATGGGCTCGCACAGGAAGATTTGTGTTACAGTCGTCATCTTTGCCTCCCCAAAAAAAAAACCCAAACGAATTAGAAGAAAAGGCGAACAAATCAAGAGTATGGAGAGTAGAAAACAGGGACAGCAGGAACCAAGACATAAGAAAGCCCCCAATACAGCAGTGCCAGGCAATAGGTAGTAGTAGATGGGTTCGCATAGAAAGGCCGTCCAATCTGTAGCAGATGGTTGCCTTTGCATGGAATACCCAAGCAGCATTTCCAATTTTCATGAGCAAACACCAGCTAGTGTATAGGTTGCCCAAATATAAGAAGCCACTATATAGGCACAGGCAGAGGGGTTGCAACCAATCAAATTGCTCACCTTACACTGTAGAGAAACAGAGAGGTTCGTACAGCTGTTTTTTTGGAGCAAATAGTACATGCTATTTATAGGCTCAGAATCACAGGATATCTTCAGGATATTTTTTCTACGAAGCTGGAACAATGATTTTTTGCAAATACAAAAGCAGACATGATGATGACAGAATAGATTTCAGGATATCTTCTCATTTATTCTAAAGATCTCAGAATATCTTTAGAATTATTTTTTAAAAGAAAGCTTCTGGCAATAGGGAATAAAGAGATGCGAAGTAAAATCTTAGGAGATCTGATGAGAGAGGAGATGAGAGAGGATTACCTGT
